# Older adults' attitudes toward using Euthanasia at the end-of life: cancer vs. Parkinson's disease

**DOI:** 10.3389/fpubh.2024.1393535

**Published:** 2024-06-14

**Authors:** Rinat Lifshitz, Yaacov G. Bachner, Sara Carmel

**Affiliations:** ^1^Community Gerontology, The Max Stern Academic College of Emek Yezreel, Emek Yezreel, Israel; ^2^Program in Gerontology, Department of Epidemiology, Biostatistics and Community Health Sciences, Ben-Gurion University of the Negev, Be'er Sheva, Israel

**Keywords:** older adults, end-of life decisions, attitudes, Euthanasia, cancer, Parkinson's disease

## Abstract

**Background:**

There is a paucity of studies that compare older adults' attitudes toward Euthanasia in two different terminal illnesses. Moreover, these studies did not relate to potentially influencing psycho-social factors. The current study aimed to examine the impact of a diverse range of variables on attitudes among older adults toward Euthanasia in two medical conditions: cancer and Parkinson's disease.

**Methods:**

A total of 501 individuals aged 75 and above participated in the study. Attitudes toward Euthanasia were measured using vignettes which described two conditions: an 80-year-old man with metastatic cancer and another man in an advanced stage of Parkinson's disease. The questionnaire included measures of relevant experience (with a close family member or a friend dying from a terminal illness), self-efficacy, will to live, satisfaction with life, will to prolong life, fear of death and dying, social support, and psycho-social characteristics. The data were analyzed using hierarchical linear regression models.

**Results:**

A more positive attitude toward Euthanasia was found in the case of cancer compared to Parkinson's disease. Being a woman, having more years of education, lower level of religiosity, greater fear of death and dying and higher self-efficacy contributes to more favorable attitudes toward Euthanasia in both end-of life conditions.

**Conclusions:**

The finding that attitudes toward Euthanasia are statistically significantly more positive in the case of cancer compared to Parkinson's disease can be attributed to the greater prevalence of cancer in the population, and to the public's awareness of the suffering associated with each of these medical conditions. Beyond the important role of the socio-demographic characteristics of gender, education, and religiosity, it appears that fear of death and dying and self-efficacy are important psychological factors in explaining attitudes toward Euthanasia in both illnesses among older people. These findings shed light on older adults' attitudes toward Euthanasia in debilitating illnesses.

## Introduction

Modern medicine makes it possible to prolong the life of patients with incurable diseases, but prolonging life is often accompanied by chronic physical and mental anguish for patients. There is little agreement about what constitutes a good death. According to the literature, the definition of a good death includes three main themes: preferences for the dying process, pain-free status, and emotional wellbeing (1). Thus, in recent years, various end-of-life options are at the focus of health care practitioners, policy makers and the public.

Active and voluntary Euthanasia (EUT) refers to the act of intentionally ending the life of a patient by means of active drug administration at his explicit request (2). In the case of physician-assisted suicide (PAS), the physician supplies the drug, but the patient administers it (3). In many countries, EUT remains a controversial topic in both public discourses and legislation. According to Statista (4), around the world, only a few countries have legalized assisted dying, but their number has been growing recently. The practice of both self-PAS and EUT have been legal in the Netherlands, Belgium and Luxembourg since the 2000s and has been practiced in Switzerland, which only allows the former, since the 1980s. Colombia legalized EUT in 2015 and PAS in 2022 and both types also became legal in Canada in 2015. Spain, Austria, New Zealand and several Australian states have made EUT and PAS legal in recent years too. In the US, PAS is legal in nine states and the District of Columbia (5). In Germany and Israel, medical treatments may be withheld under certain conditions, but while EUT is strictly forbidden under all circumstances in both countries, PAS is possible in Germany.

### Attitudes toward EUT in various conditions

Research conducted over several decades has shown that in many countries, most people support the legalization of EUT (6). In a study conducted in Israel in 1994 on a random sample of older adults aged 70+, 52% supported legislation of voluntary active Euthanasia in severe illness conditions (7). What is less clear from this research is the extent to which these attitudes vary with the type and condition of the patient's illness.

Cancer and cardiovascular diseases are the leading causes of death in developed countries (2). A Swiss study that identified trends of PAS among patients over 18 years in age (1999–2016; *n* = 6,553) found that cancer was the most common underlying disease (*n* = 2,704, 41.3% of all PAS cases) and cancer patients were considerably younger than patients with other diseases (73 years vs. 80 years) (8). Schuurmans et al. (9) report that in all countries where EUT is legalized, it primarily concerns patients with cancer. In addition, more favorable attitudes toward EUT were reported when patients had cancer in comparison to patients with mental illnesses such as schizophrenia or clinical depression (6). After cancer, the most frequent requests for EUT or PAS were from patients diagnosed with neurological diseases. Dementia, motor neuron disease, multiple sclerosis, and Parkinson's disease (PD) are the neurological diseases that most frequently motivate requests for EUT or PAS. Requests related to dementia are the most growing while raising ethical and legal issues due to these patients' diminished competent (10). Overall, the wish for EUT arises in situations of burdensome care and fear of future deterioration and suffering (11).

### Older adults' attitudes toward EUT

The increase in life expectancy on the one hand and the decline in fertility on the other hand are followed by the aging of populations in the Western world. These trends lead to an increasing number of older adults who suffer from chronic and terminal illnesses accompanied by severe disabilities and a prolonged dying process (12).

Many studies have found that older adults and those close to death would like to have the choice of ending their lives to avoid impending suffering (13, 14). In the case of EUT, studies of older American adults found that about half of them support the legalization of EUT (15). In a random sample of 1,136 Israelis aged 70 and older, participants' attitudes regarding Euthanasia were found to be more positive regarding patients who were physically ill, as opposed to patients with mental illness (16). It seems that physical illness in old age is seen as a more compelling reason for voluntary assisted death (VAD) than mental illness (12, 17).

### Factors associated with attitudes toward EUT

Over the years, numerous variables have been examined regarding their association with EUT. In this study we focused on two groups of factors: socio-demographic variables and psycho-social characteristics. While quite a bit of research has been conducted on the first group, there are few studies about the contribution of psycho-social characteristics to people's attitudes toward EUT.

#### Socio-demographic variables

The findings of studies carried out in different countries regarding the associations between socio-demographic variables and attitudes toward EUT are inconsistent. In many studies, age was found to be negatively associated with positive attitudes toward EUT, negative attitudes toward EUT were reported with more frequence among the older age groups compared to younger adults (18–21). In contrast, in an Israeli sample of people aged 40 years and over, a positive correlation was found between age and preferences for end-of-life practices, with higher support for EUT in the older group than in the younger age group. Other studies found no statistically significant correlations between age and attitudes toward EUT (22–24).

As for gender, it seems that men demonstrate more favorable attitudes toward EUT than women (3, 18, 20, 25–27). Some studies found positive correlations between education and positive attitudes toward EUT (18–21) and between socio-economic status and more favorable attitudes toward EUT (18, 19, 22, 23). People living alone mostly reported more positive attitudes toward EUT than people who lived with a partner or who had children (23, 28), and poor physical status or comorbidity correlated with more positive attitudes toward the legalization of EUT (29).

Attitudes regarding matters of life and death are often derived from one's most basic beliefs and values. Since prevalent religions prohibit all forms of euthanasia and suicide, religious beliefs are important factors in explaining attitudes toward EUT. Most studies reported that religiously observant persons displayed more negative attitudes and behaviors toward EUT and assisted suicide than secular people (5, 21, 30, 31). However, some variation exists between different religions and religious denominations (3, 18, 20, 24, 32, 33). For instance, for Israeli Jews, a positive correlation was found between religiosity and attitudes toward prolonging life and a negative correlation with attitudes toward EUT (34–37).

Previous experience with the death of someone close or a loved one due to a severe terminal disease has been shown to be a significant factor in older adults' attitudes toward end-of-life practices (33, 38–40). Vilpert et al. (21) found that previous experience as a healthcare proxy was positively associated with more favorable attitudes and behaviors toward EUT.

#### Psycho-social variables

Notably, there is a gap of knowledge regarding the possible contribution of some well-known psycho-social variables to the explanation of attitudes toward EUT. Only a few studies have focused on older adults' attitudes toward EUT (7, 33, 41) and those displayed heterogeneous findings (19). *Satisfaction with life* is considered a cognitive-judgmental component of subjective wellbeing (42) and thus was studied in the context of attitudes toward death and dying. However, findings regarding the correlations are ambiguous. Some studies reported positive correlation between satisfaction with life and attitudes toward death and dying (43), while others reported negative correlations or none (44, 45). Among Dutch community-based older adults who were functionally impaired, a favorable attitude toward assisted death (labeled *hastened death* in the study) was negatively associated with life satisfaction (46).

*Fear of death and dying* among older adults was found to correlate with attitudes toward use of life-sustaining treatments at the end of life and EUT (39). In another study, greater death anxiety was associated with a desire for more medical intervention in end-of-life scenarios (47) and less acceptance of EUT (19). *Social support* is known to be an important coping resource when facing life threatening situations (48). Numerous studies have shown that social support reduces the stress experienced by an individual (49). Studies of terminally ill patients have found that the desire to hasten death is associated with the absence or a low degree of social support (50–53). Nevertheless, in a study with ALS patients, no correlation was found between social support and the desire to hasten death by medical interventions (54).

*Self-efficacy* refers to an individual's belief in his ability to perform various actions successfully (55). This belief influences behavior and ways of coping with various stressful events throughout life. A high perceived self-efficacy was found to reduce fear of impending death for older adults (56). The *will to live* is a psychological expression of the individual's commitment to life and its desire to continue living. Will to live comprises instinctive and psychological dimensions. A national study of older Israeli adults found that the will to live is an important factor influencing the wish for more medical intervention in end-of-life scenarios (39, 57).

### The present study

Few studies exist regarding older adults' attitudes toward EUT and physician assisted suicide. Moreover, the existing studies focus mainly on a single illness (cancer or Alzheimer's) and do not include potentially influencing psycho-social factors. The goals of the current study were twofold: (1) to compare older adults' attitudes toward EUT in two illness conditions (cancer vs. PD) (2) to examine the correlations between socio-demographic and psycho-social variables and attitudes toward EUT in each of the two illness conditions.

## Materials and methods

### Sample and procedure

Five hundred and one persons aged 75 years and older participated in the study. Inclusion criteria included age 75 years or older, the ability to speak Hebrew, and being able to understand and reply to the study questionnaire. Prospective participants were recruited using a convenience sample (e.g., adult daycare centers) and snowball sampling was implemented as well. All participants were informed of the research goals and were told that participation is voluntary and anonymous. Those who agreed to participate signed an informed consent form before being interviewed by experienced interviewers. The study received ethical approval by the second and third authors' Institutional Review Boards. Table 1 presents the sociodemographic characteristics of the sample. The participants' range of age was 75–94 (*M* = 80.96, *SD* = 4.51), most of them were women, married, with tertiary education, rated their health condition as good and their economic status as average and higher.

**Table 1:** Socio-demographic characteristics of the study participants.

**Variable**	**n**	**%**
**Gender**		
Male	222	44.6
Female	276	55.4
**Education**		
Primary and secondary	242	43.8
Tertiary	251	53.2
**Economic status**		
Lower than average	63	13.9
Average and higher	416	86.1
**Family status**		
Not married	221	44.2
Married	264	53.8
**Variable**	**M (SD)**	**Range**
Age	80.96 (4.52)	75–94
Self-rated health	4.19 (1.02)	1-6
Religiosity	2.65 (1.24)	1-5

Family status was coded 1 = not married (single, widower, divorced), 2 = married; Self-rated health coded 1 = very bad to 6 = excellent; Religiosity was coded 1 = low to 5 = high.

### Measures

*Participants' attitudes toward EUT* were measured using the following vignettes which describe two conditions:

Condition A: An 80-year-old man suffers from metastatic cancer, with no chance of recovery. Doctors estimate he has about 6 months left to live. The patient suffers great physical pain and needs the help of others to perform basic activities (e.g., self-feeding, self-dressing). The patient has received explanations from his doctor about the ability to control pain and the possibility of receiving supportive treatment that relieves the physical and mental symptoms (palliative care) in a home hospice or institutional hospice setting. However, the patient has asked his physician several times to help him end his life due to the tremendous suffering caused by his disease.

Condition B: An 80-year-old man has been diagnosed with incurable PD. He is now at an advanced stage of the disease and needs the help of others to perform basic activities. He has asked his physician to help him end his life.

For each of these conditions, the respondents were asked whether “The doctor should inject the patient with a drug in a lethal dose to end his life,” and to rate their consent on a six-point Likert scale ranging from 1 (*definitely disagree*) to 6 (*definitely agree*). The final score for each condition was computed as the average of participants' ratings. The higher the score, the more positive is the attitude toward EUT.

*Past experience* was measured by the question: “Have you seen people close to you (family, friends) dying from a terminal illness?” using a dichotomic scale (yes/no).

*Self-efficacy* was measured by the General Self-Efficacy Scale [GSES; (58)] that includes ten items such as: “no matter what comes my way, I'm usually able to handle it.” The scale was created to assess the strength of an individual's belief in his or her own ability to respond to difficult life situations. Each item was rated on a four-point scale ranging from 1 (*not at all true*) to 4 (*exactly true*). The final score was computed as the average of ratings of all items. The higher the score, the higher the person's self-efficacy. Cronbach's alpha for the current study was very high (α = 0.93).

*Will to live* (WTL) was measured using the WTL scale (59, 60). The scale is based on five items asking the individual to evaluate the strength of its WTL in general, by comparing it to the WTL of others in its age group, during previous periods of its life. Each item was rated on a scale ranging from 0 (*no WTL at all*) to 5 (*a very strong WTL*). The final score was computed as the average of ratings for all five items. The higher the score, the higher the person's WTL. Cronbach's alpha in the currents study was very high (α = 0.91).

*Satisfaction with life* was assessed by six items representing respondents' degree of satisfaction with its life in general, physical health, mental ability, relations with friends, relations with family and ability to help the family (61). Each item was rated on a scale ranging from 1 (*not at all satisfied*) to 5 (*very satisfied*). The index score represents the average score of all six items. The higher the score, the higher the life satisfaction. Cronbach's alpha in the currents study was satisfactory (α = 0.78).

*Will to prolong life* refers to a person's will to prolong his life in difficult illness conditions (25). It was measured by five items representing the level of agreement with five statements such as: “I would accept any medical treatment in order to prolong my life,” “I would like to prolong my life as much as possible in any health condition.” Each item was rated on a scale ranging from 1 (*completely disagree*) to 5 (*completely agree*). The final score was computed as the average of ratings for all items. The higher the score, the stronger the person's will to prolong life (62). Cronbach's alpha in the currents study was very high (α = 0.91).

*Fear of death and dying* refers to a person's fear of death (e.g., of being forgotten, of separation, darkness, and decomposition of the body) and dying (e.g., pain, suffering, sensory loss, and humiliation when approaching the time of death) (62, 63). It was measured by 12 items such as: “I am very afraid of death” and “I am afraid of a long, slow death.” Each item was rated by a 5-point scale from 1 (*completely disagree*) to 5 (*completely agree*). The final score was computed as the average of ratings for all items. The higher the score, the higher the person's fear of death and dying. Cronbach's alpha in the current study was high (α = 0.82).

*Social support* was measured by the commonly used Multidimensional Scale of Perceived Social Support [MSPSS; (64)]. This 12-item self-reporting instrument asks respondents to evaluate how they feel regarding three sources of support: family (e.g., “I get the emotional help and support I need from my family”), friends (e.g., “My friends really try to help me”), and significant others (e.g., “There is a special person that is around when I am in need”) using a Likert scale ranging from 1 (*very strongly disagree*) to 7 (*very strongly agree*). The final score and the scores for the sub-scales were computed as the average of ratings for all/relevant items. The higher the score, the higher the person's perceived social support. Cronbach's alpha for the entire scale in the current study was very high (α = 0.93), as well as for each sub-scale: family, friends and others (α = 0.92, α = 0.95, α = 0.91 respectively).

#### Covariates

The study hypotheses controlled for main socio-demographic variables: age, gender, family status, education, religiosity, self-rated health, and self-rated economic status.

### Analytic strategy

Means, standard deviations and ranges were computed using descriptive statistics. Associations between background and study variables were computed using Pearson correlation coefficient, Spearman correlation coefficient, or Chi-Square tests depending on the variable's structures. Internal reliability was measured using Cronbach's alpha. The relative contributions of the independent variables to the explanation of the dependent variables were tested using hierarchical multiple regression analyses. Variables found to statistically significantly correlate with at least one of the dependent variables in the univariate analyses were included in the regression models in both medical conditions (cancer model and PD model). Significance level was set at *p* < 0.05 for all analyses. Data were analyzed using SPSS statistical software, PC version 25.0.

## Results

### Descriptive statistics of study variables and associations with attitudes toward EUT

Descriptive statistics of the study variables and their associations with EUT in two medical conditions are presented in Table 2. The average score for cancer was higher (*M* = 3.48, *SD* = 2.06) than for PD (*M* = 2.95, *SD* = 1.93). This difference was found to be statistically significant [*t*_(490)_ = 7.59, *p* < 0.001], indicating a more positive attitude toward using EUT in a severe cancer condition than in an advanced stage of PD.

**Table 2:** Descriptive statistics and Pearson coefficient correlations between main study variables (*N* = 501).

**Variable**	**Mean**	**SD**	**Range**	**Self-efficacy**	**Will to live**	**SWL**	**WPL**	**FDD**	**Social support**
Attitudes-cancer	3.48	2.06	1–6	0.132^**^	−0.023	−0.009	−0.316^**^	0.126^**^	−0.037
Attitudes - PD	2.95	1.93	1–6	0.070	−0.133^**^	0.014	−0.266^**^	0.103^*^	−0.011
Past experience	1.28	0.44	1–2	0.003	0.061	0.035	0.160^**^	−0.066	−0.021
Self-efficacy	3.15	0.65	1–4	1					
Will to live	3.98	0.94	0–5	0.434^*^	1				
SWL	3.93	0.75	1–5	0.568^**^	0.524^**^	1			
WPL	2.68	1.31	1–5	0.103^*^	0.247^**^	0.155^**^	1		
FDD	3.18	0.74	1–5	−0.150^**^	−0.061	−0.138^**^	0.006	1	
Social support	5.65	1.20	1–7	0.330^**^	0.279^**^	0.556^**^	0.093^*^	−0.026	1

SWL, Satisfaction with life; WPL, Will to prolong life; FDD, Fear of death and dying. ^*^p < 0.05, ^**^p < 0.001.

Satisfaction with life, self-efficacy and social support were found to be high, while the will to live, will to prolong life and fear of death and dying were found to be average (relative to the scale range). Past experience was found to be high with 71.3% reporting having had past experience with people close to them dying from a terminal disease.

In the case of cancer, attitudes toward EUT positively correlated with self-efficacy, and fear of death and dying and negatively correlated with the will to prolong life. As for PD, attitudes toward EUT were positively correlated with fear of death and dying and negatively correlated with the will to prolong life, the will to live and receiving social support from family.

A statistically significant negative correlation between age and the attitude toward EUT was found in the case of cancer (*r* = −0.111), as well as negative correlations between religiosity level and attitudes toward EUT in both cases–cancer and PD (*r* = −0.562, *r* = −0.467 respectively). Young and secular participants manifested more positive attitudes toward EUT in the case of cancer than others (Full matrix available from the first author upon request).

Relationships in attitudes toward EUT between the variables of gender, family status, education level and economic status were tested using independent *t-*test (shown in Table 3). Statistically significant differences were found between men and women in the case of cancer *t*_(478)_ = −2.134, *p* = 0.017 and in the case of PD *t*_(478)_ = −1.724, *p* = 0.043. Women reported more positive attitudes toward EUT in cancer and in PD (*M* = 3.65, *SD* = 2.09; *M* = 3.07, *SD* = 1.98 respectively) than men *(M* = 3.25, *SD* = 2.00; *M* = 2.77, *SD* = 1.83 respectively). Also, statistically significant differences were found in attitudes toward EUT between participants with different levels of education in the case of cancer *t*_(458)_ = −4.051, *p* < 0.001, and in the case of PD *t*_(461)_ = −3.133, *p* < 0.001. Older adults with tertiary education reported more positive attitudes toward EUT in cancer and in PD (*M* = 3.90, *SD* = 2.01; *M* = 3.27, *SD* = 1.85 respectively) than older adults with primary and secondary education *(M* = 3.14, *SD* = 2.05; *M* = 2.71, *SD* = 1.96 respectively). No statistically significant differences were found for family and economic status.

**Table 3:** Differences in gender, economic status and family status regarding EUT in two illness conditions: results of independent *t*- test (*N* = 501).

**Variable**	**Attitudes toward EUT - Cancer**				**Attitudes toward EUT – PD**			
	***M***	***SD***	***t***	***p***	***M***	***SD***	***t***	***p***
**Gender**								
Men	3.25	2.00	−2.134	0.017	2.77	1.83	−1.724	0.043
Women	3.65	2.09			3.07	1.98		
**Education**								
Primary and secondary	3.14	2.05	−4.051	<0.001	2.71	1.85	−3.133	<0.001
Tertiary	3.90	2.01			3.27	1.96		
**Economic status**								
Lower than average	3.12	1.98	−1.601	0.056	2.71	1.79	−1.09	0.137
Average and higher	3.53	2.07			2.97	1.93		
**family status**								
Not married	3.47	2.03	−0.337	0.368	2.88	1.87	−0.933	0.176
Married	3.53	2.10			3.05	1.92		

### Regression analyses of variables explaining attitudes toward EUT

Results of the first hierarchic regression analysis on attitudes toward EUT in the case of cancer are presented in Table 4. In the first block reflecting the participants' socio-demographic characteristics, being a woman, having higher education and having low level of religiosity emerged as statistically significant explanatory variables of attitudes toward EUT in patients with terminal cancer. The model explained 33.6% of the observed attitude variance and was found to be statistically significant, *F*_(4, 446)_ = 55.909, *p* < 0.001. The second block, to which the participants' psycho-social characteristics were added, showed a modest but statistically significant change of 2.8% in R^2^. All of the variables in the equation explained 36% of the observed variance, *F*_(9, 446)_ = 27.814, *p* < 0.001—a relatively wide range of the variability on the attitude toward EUT. In the final model, two variables emerged as statistically significant positive predictors of the attitude toward EUT in the case of cancer: Fear of death and dying β = 0.121, *p* = 0.002, and self-efficacy β = 0.116, *p* = 0.010.

**Table 4:** Factors explaining attitudes toward EUT in two conditions – cancer and PD (*N* = 501).

	**Attitudes toward EUT – Cancer**				**Attitudes toward EUT - PD**			

	***B***	***S.E***	***Beta***	*△**R**^2^*	***B***	***S.E***	***Beta***	*△**R**^2^*
**Step 1**				0.336				0.24
Age	0.002	0.014	0.006		0.011	0.014	0.035	
Gender	0.533	0.161	0.129^***^		0.48	0.162	0.124^**^	
Education	0.488	0.164	0.118^**^		0.408	0.165	0.105^*^	
Religiosity	−0.811	0.06	−0.538^***^		−0.641	0.061	−0.454^***^	
**Step 2**				0.028				0.039
Age	0.004	0.014	0.012		0.007	0.011	0.022	
Gender	0.468	0.16	0.113^**^		0.415	0.16	0.107^*^	
Education	0.426	0.167	0.103^*^		0.389	0.167	0.100^*^	
Religiosity	−0.759	0.066	−0.503^***^		−0.636	0.066	−0.451^***^	
Fear of death and dying	0.344	0.111	0.121^**^		0.221	0.11	0.083^*^	
Will to prolong life	−0.123	0.068	−0.079		−0.011	0.68	−0.008	
Will to live	−0.169	0.098	−0.078		−0.403	0.098	−0.199^***^	
Self-efficacy	0.382	0.147	0.116^*^		0.36	0.147	0.117^*^	
Social support from family	0.001	0.064	0.001		−0.016	0.064	−0.011	
Model F	27.814^***^				18.599^***^			
R squared				0.364				0.279

Gender was coded 1 = man, 2 = woman; Education was coded 1 = elementary and secondary, 2 = tertiary.

Religiosity was coded 1 = low to 5 = high. ^*^p < 0.05, ^**^p < 0.01, ^***^ *p* < 0.001.

Results of the second hierarchic regression analysis on the attitude toward EUT in the case of PD are presented in Table 4. In the first block presenting the participants' characteristics, being a woman, having higher education and having a low level of religiosity emerged as statistically significant predictors of attitudes toward EUT in PD. The model explained 24% of the attitude's variance and was found to be significant, *F*_(4, 441)_ = 34.478, *p* < 0.001. The second block, in which the participants' psycho-social characteristics were added, resulted in a modest but statistically significant change of 3.9% in R^2^. All of the variables in the equation explained 27.9% of the observed variance, *F*_(9, 441)_ = 18.599, *p* < 0.001. In the final model, three variables emerged as statistically significant explanatory variables for attitudes toward EUT in PD: Fear of death and dying β = 0.083, *p* = 0.046; self-efficacy β = 0.117, *p* = 0.015 and will to live β = −0.199, *p* < 0.001.

## Discussion

The current study contributes several important insights to our understanding of the role of socio-demographic characteristics and psycho-social factors in explaining attitudes of older adults toward EUT for two terminal medical conditions: metastatic cancer and advanced stage of PD. Some of our findings support previous research while others are new. Overall, we found a positive tendency among older adults toward using EUT in both conditions. This finding is aligned with past results that demonstrate the public's support of legalization of active euthanasia in end-of-life situations in different age groups (6, 7, 15). It should be noted that some studies mention that the use of case studies favors the acceptance of euthanasia and physician-assisted suicide among people, compared to when general norms are presented (65). Yet, attitudes toward EUT in the case of cancer were statistically significantly more positive than in the case of PD. In addition, the variability in participants' attitudes toward EUT was greater in the case of PD and the explaining power of our model was found to be weaker in the case of PD.

These differences may be explained by *illness perceptions* of the two types of diseases—cancer and PD. Illness perception refers to organized beliefs regarding the symptoms, consequences, time course, controllability, and causes of an illness (66, 67). Most studies focus on illness perception as perceived by the chronically ill patients themselves, rather than on the general public's perceptions. Additionally, vast research has been carried out in the case of cancer since metastatic cancer is perceived as associated with the highest burden on mental and physical health-related quality of life (68). Consequently, due to the wide prevalence and publicity of this type of cancer and how it is perceived, the finding in the current study pointing at older adults' stronger positive attitude toward EUT in metastatic cancer in comparison to PD, is understandable.

Regarding PD, our finding of lower support for EUT is somewhat surprising since the deterioration in cognitive and emotional functions and speech disorders which affect many patients at advanced stages of PD significantly influence their quality of life, as well (69).

This inconsistency may be explained by former studies claiming that cancer, more so than neurological diseases, is the most frequent cause for requests for EUT, probably due to cancer's high prevalence in the population and the elevated public awareness to this disease (9, 10). Specifically, cancer is more prevalent than PD among older adults. With the aging of the population, there will be a considerable increase in the number of older adults diagnosed with cancer (70). Moreover, Moore and Knowles (71) reported that negative attitudes to PD are associated with perceived stigma and younger age. In our study, older adults seem to be more concerned with being ill with cancer than with PD. A reason for this might be that PD was not perceived as a terminal illness.

The associations found between socio-demographic characteristics and attitudes toward EUT were partly inconsistent with previous studies. While most studies report more favorable attitudes toward EUT among men compared to women (3, 18, 20, 25–27), we found that older woman exhibit more positive attitudes toward EUT than men. Our finding is in line with previous findings in Israel regarding wishes for the prolongation of life which were found to be higher among men than among women (72), and women's weaker will to live than that of men (25). Additionally, participants with higher level of education and lower level of religiosity manifested more positive attitudes toward EUT in both medical conditions. These findings support former studies which found positive correlations between education levels and attitudes toward EUT (18–21), and negative correlations between religiosity level and favorable tendency toward EUT (5, 21, 30, 31, 34, 37).

Although numerous studies have investigated attitudes toward EUT, little is known about the impact of psycho-social characteristics on older adults' perceptions. Our findings reveal similarities for both medical conditions whereas two well-known factors, fear of death and dying and self-efficacy, were found to be positively associated with a more favorable attitude toward EUT. Although these findings conform with some previous studies (39, 56), contradicting results were found in a recent study regarding death anxiety in various end-of-life scenarios (19, 47). Since our sample consisted of individuals aged 75 years and older, and most of them (71.3%) reported having experience with people close to them dying from a terminal illness, death seems to be perceived among them as part of life. This supports previous reports (13, 39), indicating that older participants were less worried about death itself, while very concerned about the dying process (the way to death).

An in-depth comparison of the psycho-social data between the two illness conditions reveals an interesting finding. Although fear of death and dying and self-efficacy were found to positively correlate with attitude toward EUT in both conditions, they were more significant contributors in the case of cancer than in the case of PD.

Most studies regarding fear of death and dying and EUT focused on patients with advanced cancer, because their symptoms often have a large impact on quality of life and end-of-life care (73). Less is known about the impact of these factors on attitudes toward EUT in the case of PD. Considering the rise in latest years in life-threatening neurological diseases (74), the impact of psychosocial variables on people's perceptions of such diseases, as well as the derived attitudes and social norms should be further studied.

Self-efficacy, an individual's belief in its ability to perform various actions successfully (55), was found in multivariate analyses to be another statistically positive significant explanatory variable of attitudes toward EUT. Literature indicates that self-efficacy reduces fear of impending death (56) and psychological distress (75) among older adults. This can explain the positive correlation found in this study between self-efficacy and attitudes toward EUT. It is possible that people with a strong feeling of self-efficacy support EUT to avoid unpleasant psychological situations. Furthermore, as metastatic cancer is probably perceived by older people as a more deteriorating disease than PD, the attitude toward EUT in cancer may be more affected by one's self-efficacy than in PD.

As expected from a previous study (39), the will to live negatively contributed to the will for EUT intervention but only in the case of PD. Among dying patients, will to live shows substantial fluctuation, with the explanation for these changes shifting as death approaches (76). It seems that PD is perceived by the public as a slow deteriorating disease and less aggressive than cancer, thus higher will to live was found in the study to correlate with lower attitudes toward EUT.

A major conclusion of our study is that older adults present a tendency for favorable attitudes toward EUT in two different severe illness conditions. However, the attitude for EUT in the case of cancer is significantly more positive than in the case of PD. This pattern can be attributed to the prevalence of these illness conditions, which is higher for cancer than for PD, and to the public's awareness regarding the suffering associated with the deterioration stages in each condition. In conclusion, beyond the important role of the socio-demographic characteristics of gender, education, and religiosity, it appears that fear of death and dying and self-efficacy are important psychological factors in explaining attitudes toward EUT in both cancer and PD among older people.

### Limitations and future research

Our findings shed some light on the contribution of socio-demographic and psycho-social variables to the explanation of attitudes concerning EUT in two severe illnesses that differ in prevalence and in public awareness regarding end-stage quality of life.

Nevertheless, the study embodies several drawbacks. First, the design was cross-sectional, which does not allow one to trace changes in attitudes and to make causal interpretations. Moreover, the sample consisted of older Jewish adults who were highly educated and at a relatively high economic status. Considering these characteristics and the sampling method employed, the sample is not representative of the Israeli older population. Additionally, a significant part of the recruitment process was performed during the COVID-19 crisis period which may have influenced the participants' attitudes in one way or another.

Future research on attitudes toward EUT among various groups of older adults is warranted and should be expanded to include additional factors such as personality characteristics, cultural values, beliefs, and societal norms of behavior. Studies should also use qualitative methods to gain a deeper understanding of the factors that shape attitudes toward EUT. All of these will hopefully provide more insights into the mechanism that regulates older adults' decision processes regarding end-of-life treatments, including EUT.

## Data availability statement

The original contributions presented in the study are included in the article/supplementary material, further inquiries can be directed to the corresponding author.

## Ethics statement

The studies involving human participants were reviewed and approved by the Faculty of Health Sciences at Ben-Gurion University of the Negev, Beer-Sheva, Israel. The patients/participants provided their written informed consent to participate in this study.

## Author contributions

RL: Conceptualization, Data curation, Visualization, Writing – original draft, Writing – review & editing. YB: Conceptualization, Writing – review & editing. SC: Conceptualization, Supervision, Writing – review & editing.
